# Anticipated Stigma in Nursing: A Concept Analysis Informed by Cannabis Use Disclosure

**DOI:** 10.1111/jan.70499

**Published:** 2026-01-26

**Authors:** Daniel D. King

**Affiliations:** ^1^ College of Nursing Rosalind Franklin University of Medicine and Science North Chicago Illinois USA

**Keywords:** anticipated stigma, cannabis, concept analysis, disclosure, nurse–patient relations, nursing, therapeutic communication

## Abstract

**Aim:**

To clarify the concept of anticipated stigma and examine its relevance to cannabis use disclosure in nursing using an evolutionary concept analysis approach.

**Design:**

Concept analysis guided by Rodgers and Knafl's evolutionary method.

**Data Sources:**

An interdisciplinary purposive literature review was conducted using empirical and theoretical sources drawn from nursing, public health, psychology and sociology. Literature published between 1963 and 2024 was included, with specific emphasis on health‐related stigma, disclosure and cannabis use.

**Review Methods:**

Rodgers and Knafl's evolutionary method was used to identify the defining attributes, antecedents, consequences, related concepts and contextual variations of anticipated stigma. Empirical and conceptual literature were synthesised to reflect cross‐disciplinary themes. A model case was constructed to illustrate the concept's application in a nursing context, followed by a critical synthesis of implications for nursing theory, research and practice.

**Results:**

Anticipated stigma is a future‐oriented expectation of devaluation or discrimination associated with disclosing a stigmatised identity or behaviour. Five core attributes were identified. Antecedents include individual identity salience, sociocultural norms and structural factors. Consequences include psychological distress, concealment and reduced healthcare engagement.

**Conclusion:**

Anticipated stigma is a dynamic and under‐theorised concept that hinders therapeutic communication and person‐centred care in nursing settings.

**Impact:**

This analysis offers conceptual clarity and supports stigma‐informed approaches to assessment and communication in nursing education and practice, especially when addressing cannabis use and other stigmatised health behaviours.

**Patient or Public Contribution:**

No patient or public contribution.

## 1 Introduction

Anticipated stigma refers to a future‐oriented expectation of prejudice, devaluation, discrimination or negative judgement that may occur if a concealable stigmatised identity, condition or behaviour is disclosed. This anticipation is shaped by awareness of societal stigma and can negatively affect psychological well‐being, disclosure decisions, help‐seeking and engagement in care (Earnshaw, Smith, et al. 2013; Kane et al. 2019; King et al. 2024). Anticipated stigma has been documented across diverse countries and health systems in relation to HIV, mental illness, chronic illness and other conditions, accentuating its relevance for nurses working in global and multicultural contexts (Fujii and Hashimoto 2023; Ikizer et al. 2018; Liu et al. 2020; Peltzer and Pengpid 2016).

In this concept analysis, anticipated stigma is operationalised as an expectation of devaluation or mistreatment specifically in health and social contexts, with particular attention to cannabis use disclosure. Persistent worry about potential mistreatment leads many patients to conceal stigmatised behaviours, such as cannabis use, even when disclosure is essential to provide informed, safe care. Because nurses often serve as the primary point of contact for assessment, education and ongoing support, they are uniquely positioned to recognise manifestations of anticipated stigma, respond to disclosures and influence the level of psychological safety that patients experience in healthcare settings (Amarante et al. 2024; King et al. 2024; Rice et al. 2019).

Although clinically significant, anticipated stigma remains under‐theorised, particularly in nursing. Much of the existing work has been grounded in social and health psychology, drawing on frameworks such as Goffman's stigma theory, the Minority Stress Model and the Conservation of Resources Theory. These perspectives help explain how anticipated stigma arises from everyday interactions and structural inequalities, but their implications for nursing assessment, communication, and policy remain poorly articulated. Using Rodgers and Knafl's (2000) evolutionary concept analysis method, this paper traces the historical development of anticipated stigma, identifies its key attributes, antecedents, and consequences, and differentiates it from related constructs. A model case illustrates its relevance to nursing practice, with particular emphasis on cannabis use disclosure, and implications for future research, education and policy are discussed. Generative AI technologies supported organisation, synthesis of literature, and refinement of language and structure during manuscript preparation. All content was reviewed and finalised by the author.

## 2 Background

### 2.1 Historical Evolution

Anticipated Stigma has evolved from early ideas of moral shame to a more complex and layered concept with broad applications in healthcare and the social sciences (Herek 2007; Herek et al. 2009; Reid 2020). Before the 20th century, stigma was primarily associated with religious and moral transgressions, laying the groundwork for sociological and psychological exploration by the mid‐20th century. By 1963, a major shift occurred when Canadian–American sociologist Erving Goffman published *Stigma: Notes on the Management of Spoiled Identity*. Goffman introduced key concepts such as discredited (visible) versus discreditable (concealable) identities and normification (presenting oneself as normal). These ideas significantly shaped our understanding of stigma in social interactions and healthcare (Bottorff et al. 2013; Reid 2020).

During the 1990s, discussions of mental health and HIV‐related discrimination began identifying expectations of rejection and anticipated discrimination as important stigma mechanisms (Link et al. 1997; Markowitz 1998). A pivotal shift occurred in 2009 when Earnshaw and Chaudoir introduced their HIV Stigma Framework. They clearly described anticipated stigma as a subdomain of stigma, distinct from enacted and internalised stigma, and this framework has been applied across psychology, public health and medicine. In the 2010s and 2020s, anticipated stigma has been increasingly measured in contemporary healthcare research using condition‐specific tools to study disclosure, provider communication and health outcomes (Bottorff et al. 2013; Earnshaw et al. 2012; Hulaihel et al. 2023; King et al. 2024). Building on Goffman's insights, anticipated stigma has become a more acknowledged barrier to disclosing concealable stigmatised identities like cannabis use during clinical encounters (Bottorff et al. 2013; Hulaihel et al. 2023; King et al. 2024).

New frameworks have also emerged to incorporate structural stigma, intersectionality and source‐specific variations (Nelson 2021; Reid 2020; Amarante et al. 2024), with research highlighting the unequal impact on marginalised populations. Across various fields, especially in public health and psychology, there has been a growing focus on stigma's effects on care quality, mental health and behaviour. While the social sciences continue to explore concepts such as normification and cultural embeddedness, in nursing, both theoretical progress and empirical studies on anticipated stigma remain limited. This is especially significant given nursing's core emphasis on therapeutic communication, trust and relational care. Nurses are uniquely positioned to either perpetuate or reduce stigma, particularly during sensitive disclosures like cannabis use (Bottorff et al. 2013; King et al. 2024). This gap in nursing scholarship underscores the need for further development of theoretically grounded, stigma‐informed care models.

For nursing, these developments carry significant implications. Anticipated stigma directly influences core nursing tasks such as therapeutic communication, building trust and shared decision‐making. Nurses often perform medication reconciliation, preoperative assessments and psychological screenings, during which patients may consider whether it is safe to disclose stigmatised behaviours like cannabis use. Recognising anticipated stigma as a distinct and modifiable mechanism can help nurses understand when nondisclosure is driven by fear of judgement rather than a lack of information. It can also guide the development of assessments and interventions that promote psychological safety and patient autonomy (Amarante et al. 2024; Bottorff et al. 2013; King et al. 2024; Peltzer and Pengpid 2016; Quinn et al. 2014; Rice et al. 2019).

### 2.2 Identification of Issues

Throughout its evolution, anticipated stigma has been difficult to clearly distinguish from perceived, enacted and internalised stigma, particularly when these processes co‐occur in complex behavioural contexts (Earnshaw, Smith, et al. 2013; Kane et al. 2019). In this analysis, anticipated stigma is defined as the expectation of future devaluation, judgement, or discrimination if a stigmatised identity or behaviour, such as cannabis use, is disclosed (King et al. 2024; Smith et al. 2016). This differs from perceived stigma, which reflects general awareness of societal disapproval, from enacted stigma, which involves actual experiences of discrimination, and from internalised stigma, which consists of incorporating stigmatising beliefs into one's self‐concept (Goffman 1963; Catona et al. 2016; Hathaway et al. 2011; Livingston and Boyd 2010). In King et al.'s (2024) exploratory study on cannabis disclosure in healthcare, anticipated stigma emerged as a statistically significant predictor of nondisclosure, highlighting its unique psychological burden. Participants reported fears that healthcare providers would treat them differently, not listen to their concerns, or look down on them—all core indicators of anticipated stigma. This stigma domain not only affects disclosure behaviour but has also been linked to worse mental and physical health outcomes in more studied populations, such as people living with HIV (Earnshaw, Smith, et al. 2013). Such findings should lead to further clarification and distinction of anticipated stigma, especially in nursing and healthcare settings where therapeutic relationships depend on open and transparent communication.

To build upon definitional clarity, a comprehensive framework informed by available studies on chronic illness, HIV, mental illness and other concealable stigmatised identities, has revealed seven core dimensions of anticipated stigma: (1) expected prejudice or discrimination, (2) social devaluation, (3) fear of relational distancing, (4) negative healthcare experiences, (5) institutional stigma, (6) internalisation of negative stereotypes, and (7) anticipation of public stigma (Earnshaw et al. 2012; Peltzer and Pengpid 2016; Quinn and Chaudoir 2009; Quinn et al. 2015). The salience of each dimension may fluctuate based on the visibility of the stigmatised condition, sociocultural norms and the structural characteristics of the healthcare system (Akatukwasa et al. 2021; Golub and Gamarel 2013; Quinn and Chaudoir 2009). Each dimension also holds particular significance for individuals who use cannabis, especially in the context of prevailing stereotypes and moral judgements about substance use. Additionally, anticipated stigma has been associated with poorer physiological outcomes and a lower likelihood of seeking health care (Quinn and Chaudoir 2009). During clinical encounters, patients may worry about provider judgement and confidentiality, concerns that can lead to nondisclosure of cannabis use (King et al. 2024).

Attempts to quantify anticipated stigma to predict and prevent poor outcomes have proven especially challenging due to the absence of a gold‐standard measurement tool, which has several key implications for research (Kalichman et al. 2023; Karamouzian et al. 2017; Radusky et al. 2017). Proposed, adapted and even validated scales often employ overlapping or ambiguous items across different types of stigma (e.g., perceived, internalised) or health conditions (Docksey et al. 2022; Huang et al. 2022; King et al. 2024; Radusky et al. 2017). Thus, items often appear indistinguishable across surveyed constructs. Both the meaning and measurement of anticipated stigma also vary by health condition, cultural context and intersectional identities (Akatukwasa et al. 2021; Kalichman et al. 2023; Turan et al. 2017). This lack of clear conceptual boundaries in stigma research weakens construct validity and hinders the comparability of results across studies. Serial mediation models can complicate distinguishing between different constructs; however, this can be addressed with context‐sensitive and condition‐specific frameworks. While attempting to be measured objectively, qualitative findings often reveal that rigid typologies fail to capture the dynamic nature of lived experiences with stigma (Akatukwasa et al. 2021).

As anticipated stigma is a more recent concept, it has seldom been fully defined or thoroughly examined, especially within nursing. However, interdisciplinary frameworks offer valuable and diverse insights into its complexity. Initially, within the realms of psychology and sociology, Goffman (1963) viewed stigma as a discrediting trait that reduces individuals to ‘spoiled identities’, emphasising the self‐management of social character through norms and interactions. Building on this, sociological researchers Link and Phelan (2001) outlined a stigma framework defined by labelling, stereotyping, separation, status loss and discrimination, all of which are rooted in power dynamics. Public health figures Parker and Aggleton (2003) expanded this view, seeing stigma as a mechanism that reproduces social hierarchies and inequalities rather than just individual bias. From an evolutionary perspective, psychologists Kurzban and Leary (2001) suggested that stigma evolved to protect individuals from untrustworthy or contagious others, avoiding costly interactions. Aslam and Sarwat (2021) added that anticipated stigma depletes these resources, leading to anxiety, depression and lower well‐being. This aligns with Hobfoll's (1989) Conservation of Resources Theory, which describes stigma, especially anticipated stigma, as a chronic stressor that threatens psychological resources.

Newer models from minority stress, mental health and chronic disease research explain how stigma‐related stress disproportionately impacts marginalised groups, increasing psychological distress (Quinn et al. 2014). Recent frameworks, like those by Earnshaw, Quinn, et al. (2013), emphasise the chronic, source‐specific nature of stigma and its impact on health behaviours across various settings, including healthcare, work and family. Overall, these perspectives demonstrate how anticipated stigma can be influenced in different ways—from symbolic interactionism and social cognition to structural inequality and evolutionary strategies—highlighting the need for a multi‐theoretical, context‐aware approach.

## 3 Methods

### 3.1 Design and Analytical Approach

A concept analysis of anticipated stigma was conducted using Rodgers and Knafl's (2000) evolutionary method to clarify its defining attributes, antecedents, consequences and related concepts. This method views concepts as dynamic and context dependent and proceeds through six iterative strategies: (1) identifying the concept of interest and associated terms; (2) selecting an appropriate sample of literature for data collection; (3) extracting data relevant to attributes, antecedents and consequences; (4) analysing and summarising these data; (5) identifying an exemplar or model case; and (6) proposing implications for further development. Guided by a purposive review of interdisciplinary literature, the analysis synthesised empirical and theoretical sources from nursing, psychology, sociology and public health to identify recurring themes, contextual variations and overlaps with related stigma constructs. A model case is presented to illustrate the concept in application, followed by a critical synthesis of implications for nursing theory, research and practice. In line with EQUATOR Network recommendations for interpretive and conceptual research, this manuscript adheres to the *Standards for Reporting Qualitative Research (SRQR)* checklist (O'Brien et al. 2014), which is submitted as Supporting Information. Data S1.

### 3.2 Search Strategy and Data Sources

To identify literature relevant to anticipated stigma, an iterative search strategy consistent with the evolutionary nature of Rodgers and Knafl's method was used. Initial searches were conducted using Elicit (Ought Inc.), an AI‐supported research assistant that performs semantic searches across large bibliographic corpora (including Semantic Scholar, PubMed and OpenAlex). For each search, a natural language query was entered to focus on a particular aspect of anticipated stigma, including: (a) definitions and descriptions of anticipated stigma; (b) dimensions and components of anticipated stigma; (c) theoretical perspectives and historical evolution; (d) antecedents and consequences; and (e) challenges in distinguishing anticipated stigma from related concepts.

Using Elicit, eight research queries yielded an initial pool of 2189 records from a corpus of more than 126 million academic papers. From these, 151 titles and abstracts judged most relevant to anticipated stigma in health or healthcare contexts were exported to Zotero for screening by the author. To assess the completeness of the Elicit‐assisted search, supplementary keyword searches in PsycINFO and CINAHL using the terms ‘anticipated stigma’ and ‘anticipatory stigma’ were conducted; these searches did not identify additional conceptual or empirical papers that met the inclusion criteria beyond those retrieved via Elicit and citation chaining. To ensure representation of key disciplinary perspectives and nursing‐relevant contexts, the search was further supplemented through citation chaining of included articles and incorporation of seminal sources from the author's prior integrative reviews on stigma and cannabis disclosure.

### 3.3 Eligibility Criteria and Study Selection

Sources were included if they: (1) used the term *anticipated stigma* (or closely related terms such as *anticipated discrimination* or *expected stigma*) as a primary construct or clearly defined variable; (2) examined anticipated stigma in relation to health, illness, disclosure, or healthcare interactions; and (3) were empirical studies, theoretical papers, concept analyses, or narrative reviews published in peer‐reviewed journals. Sources that discussed stigma only in general terms without distinguishing anticipated stigma from perceived, enacted or internalised stigma were excluded, as were commentaries, editorials, conference abstracts and dissertations.

Titles and abstracts identified were screened against these criteria, followed by full‐text review of potentially relevant papers. After deduplication, 89 unique records were screened; 31 met the inclusion criteria and contributed directly to the concept analysis. Characteristics of these 31 sources are summarised in Table 1. An additional 34 sources were identified through citation chaining and prior integrative reviews. They were included when they provided necessary conceptual or theoretical clarification (e.g., Goffman's foundational work, stigma frameworks and nursing‐specific applications). The literature included in the final analysis spanned the years 1963 to 2024.

**TABLE 1:** Characteristics of included sources informing the concept analysis of anticipated stigma (*n* = 31).

Author & year	Discipline/field	Population and context	Health condition/focus	Source type	Term/measurement of anticipated stigma	Main conceptual contribution
Akatukwasa et al. (2021)	Infectious diseases/public health/sociology	Rural communities in Kenya and Uganda, baseline year of a large HIV test‐and‐treat trial (SEARCH)	HIV	Qualitative empirical	Anticipated stigma (narratives/qualitative themes, including fears of marital dissolution, verbal and physical abuse, gossip and public ridicule)	Links anticipated stigma to care avoidance, care‐seeking at remote facilities, and hiding of HIV medications. Highlights anticipated stigma as gendered and salient for women who experienced enacted stigma from partners
Amarante et al. (2024)	Public health/medicine/psychiatry	Transgender women (TGW) living with HIV, São Paulo metropolitan area, Brazil	HIV/transphobia/gender identity stigma	Quantitative empirical (mediation analysis)	Anticipated stigma (fear of public mistreatment due to gender identity; single binary item derived from Likert scale)	Links anticipated stigma significantly to communication difficulties with healthcare providers. Suggests structural factors (unemployment, sex work, name change issues) act as antecedents to anticipated stigma
Aslam and Sarwat (2021)	Organisational behaviour/management	Employees working in diverse organisations, South Punjab, Pakistan	Concealable stigmatised identity (CSI) (general, unspecified identity)	Quantitative empirical (survey, time‐lagged design)	Anticipated stigma (anticipated stigma; 13‐item scale adapted from prior research)	Links anticipated stigma negatively to psychological well‐being, partially mediated by higher levels of anxiety and depression. Applies Conservation of Resources (COR) theory to explain anticipated stigma as a stressor draining resources
Bottorff et al. (2013)	Nursing/public health/sociology	Individuals using cannabis for therapeutic purposes (CTP), Canada	Chronic illness (mixed, CTP focus, e.g., HIV/AIDS, fibromyalgia)	Qualitative empirical (descriptive study)	Perceived stigma (qualitative themes including anticipated labelling as ‘pothead’ or ‘criminal’)	Describes three major sources of stigma: negative view as a recreational drug, legal prohibition, and layering with existing vulnerabilities (e.g., HIV/AIDS). Identifies covert use (‘keeping it undercover’) as a primary strategy for managing anticipation
Chaudoir and Quinn (2016)	Psychology/social psychology	Emerging adults (college students) living with a concealable stigmatised identity (e.g., mental illness, sexual minority status), USA	Concealable stigmatised identities (mixed)	Quantitative empirical (longitudinal: HLM analysis)	Anticipated stigma (anticipated stigma; 15‐item multi‐item scale)	Provides longitudinal evidence that increases in anticipated stigma predict poorer trajectories of depressive symptoms (slower rate of decline) over time, independent of enacted stigma
Docksey et al. (2022)	Health psychology/organisational psychology	Employees of a large UK government organisation	Mental health problems (anxiety and depression)	Quantitative empirical (psychometric study/survey)	Anticipated stigma (anticipated stigma; 6‐item subscale of the SASS: ‘belief that other people will discriminate against you…’)	Links anticipated stigma positively to absenteeism from the workplace. Notes that anticipated stigma is defined as the belief that others will discriminate against you if you have a mental health problem
Earnshaw et al. (2012)	Psychology/health psychology	Adults living with chronic illnesses (mixed), USA	Chronic illness (mixed, e.g., epilepsy, MS, inflammatory bowel disease)	Quantitative empirical (path analysis/survey)	Anticipated stigma (CIASS subscales differentiating sources: friends/family, work colleagues, healthcare workers)	Highlights the importance of differentiating sources of anticipated stigma and demonstrating how stress, social support, and patient satisfaction act as mediators through which source‐specific anticipated stigma impacts quality of life
Earnshaw, Quinn, et al. (2013) (CIASS development)	Behavioural medicine/psychology	Adults living with chronic illnesses (students and community adults), USA	Chronic illness (mixed/generic)	Quantitative empirical (scale development/psychometric evaluation)	Anticipated stigma (Chronic Illness Anticipated Stigma Scale [CIASS]; 12‐item multi‐item scale with three subscales by source)	Develops and validates the CIASS, differentiating anticipated stigma by sources (friends/family, work colleagues, healthcare workers). Conceptualises anticipated stigma as a chronic strain and links it to decreased care access
Earnshaw, Smith, et al. (2013) (HIV stigma mechanisms)	Public health/psychology (HIV focus)	People living with HIV (PLWH), inner‐city clinic (Bronx, NY, USA)	HIV	Quantitative empirical (test of a framework)	Anticipated HIV stigma (anticipated HIV stigma; 9‐item scale, measures expectation of discrimination/stereotyping/prejudice)	Empirically tests the HIV Stigma Framework. Hypothesises that anticipated stigma, acting as a chronic stressor, predicts poorer physical outcomes (e.g., greater likelihood of chronic illness comorbidity)
Follmer et al. (2022)	Organisational behaviour/management	Employees in the United States diagnosed with depression and/or bipolar disorder, working ≥ 20 h/week	Mental illness (depression and bipolar disorder) stigma in the workplace	Quantitative empirical (longitudinal moderated mediation study)	Anticipated discrimination at work due to mental illness (9‐item anticipated discrimination scale; expectations of negative work outcomes if diagnosis is known)	Shows that anticipated discrimination about mental illness reduces authenticity at work, which in turn increases counterproductive work behaviours, and that these effects are stronger for employees with more severe symptoms, extending anticipated stigma concepts to workplace behaviour and identity processes
Fujii and Hashimoto (2023)	Public health	Middle‐aged community residents (general population), metropolitan Tokyo, Japan (during 3rd wave of COVID‐19)	COVID‐19	Quantitative empirical (cross‐sectional survey)	Anticipated stigma (single‐item question: ‘How much do you worry that you and your family will be socially excluded and/or discriminated against if you develop COVID‐19?’)	Finds perceived risk of infection and strong normative beliefs (concerning protective behaviours) independently associated with higher anticipated stigma. Suggests a dilemma where public health messages promoting adherence may inadvertently increase anticipated stigma
Golub and Gamarel (2013)	Psychology/public health/behavioural research	HIV‐negative men who have sex with men (MSM) and transgender women, New York City, USA	HIV/HIV testing behaviours	Quantitative empirical (cross‐sectional survey)	Anticipated HIV stigma (anticipated HIV stigma; abbreviated 7‐item scale focusing on expected negative consequences upon seroconversion)	Links anticipated HIV stigma negatively to regular HIV testing behaviours. Suggests anticipated stigma acts as a barrier to testing independent of perceived risk
Hathaway et al. (2011)	Criminology/sociology	Cannabis users recruited from the general adult population, metropolitan Toronto, Canada	Substance use/cannabis	Qualitative empirical (in‐depth interviews/theorising)	Stigma/internalised stigma (qualitative narratives describing fear of disclosure, labels like ‘pothead’)	Conceptualises management of stigma using Goffman's framework. Argues users reinforce cultural requirement of self‐control and engage in ‘normification’ to manage anticipated stigma and avoid the appearance of deviance
Hulaihel et al. (2023)	Nursing/public health/medicine	Patients (licensed by Ministry of Health) using medical cannabis for chronic pain, Israel	Medical cannabis/chronic pain	Qualitative empirical (phenomenological study)	Felt stigma (felt stigma: internal experience of expectation/fear of encountering enacted stigma)	Finds felt stigma is more prevalent than enacted stigma and that pre‐existing felt stigma can delay treatment initiation. Shows patients use ‘othering’ (dissociating from recreational users) and ‘normification’ to manage felt stigma
Ikizer et al. (2018)	Psychology/cross‐cultural psychology	Individuals with concealable stigmatised identities, sampled from the United States and Turkey	Concealable stigmatised identities (mixed)	Quantitative empirical (cross‐sectional survey)	Anticipated stigma (anticipated stigma; 15‐item multi‐item scale)	Examines cross‐cultural variation. Finds Turkish participants (collectivist culture) report higher anticipated stigma and depression. Anticipated stigma partially mediates the effect of culture on depression
Kalichman et al. (2023)	Psychology/public health/sexual health	Black sexual minority men (BSMM) at risk for HIV, USA	HIV prevention/intersectional stigma (race & sexual minority status)	Quantitative empirical (psychometric study/survey)	Anticipated stigma (17‐item scale with attribution ratings; geometric intersectional stigma score)	Develops a novel geometric approach to measure intersectional anticipated stigma (attributed to race and sexual minority status). Finds anticipated stigma predicts perceived barriers to using Pre‐exposure Prophylaxis (PrEP)
Karamouzian et al. (2017)	Public health/HIV research	Young people living with HIV (10–24 years), global literature review across eight countries	HIV‐related stigma among youth living with HIV	Quantitative review of empirical studies/measurement review	HIV‐related stigma measures including anticipated, enacted, internalised and perceived stigma; items coded using Earnshaw and Chaudoir's framework	Reviews how HIV stigma measures capture anticipated stigma among YLHIV and shows sex‐ and gender‐based differences are rarely examined, highlighting important gaps in measurement and gendered patterns of stigma
King et al. (2024)	Harm reduction/nursing/medicine	Adults (age 21+) using cannabis who accessed healthcare system, USA	Cannabis use disclosure/substance use	Quantitative empirical (descriptive exploratory design)	Anticipated stigma (anticipated stigma; six items adapted from SU‐SMS/multi‐item scale)	Links anticipated stigma significantly to nondisclosure of cannabis use in the healthcare setting. Finds anticipated stigma scores were the highest among the four stigma domains tested
Liu et al. (2020)	Public health/medicine (HIV focus)	HIV‐negative men who have sex with men (MSM), recruited via gay mobile app, China	HIV	Quantitative empirical (cross‐sectional survey)	Anticipated HIV stigma (anticipated HIV stigma; 7‐item Likert scale)	Links higher anticipated stigma to using social media to seek sexual partners. Links lower anticipated stigma to HIV self‐testing (HIVST) and disclosure of sexual orientation to healthcare providers
Moore and Tangney (2017)	Psychology/social psychology/criminology	Male jail inmates/offenders reentering the community, USA	Concealable stigmatised identity (criminal justice system involvement/criminal record)	Quantitative empirical (longitudinal: Structural equation modelling)	Anticipated stigma (Anticipated stigma; 4‐item adapted discrimination scale focusing on expectations)	Establishes a longitudinal model: Anticipated stigma predicts social withdrawal, which then predicts mental health problems. Identifies social withdrawal as a maladaptive coping mechanism mediating the link between anticipated stigma and poor adjustment
Ngige and Ndayala (2020)	Health/public health (HIV focus)	Adults living with HIV (PLWHA), Nairobi, Kenya	HIV self‐disclosure	Quantitative empirical (survey/logistic regression, used FGDs)	Anticipated stigma and discrimination (survey using 3‐point Likert scale on expected negative outcomes from various social networks)	Finds anticipated stigma/discrimination significantly and negatively predicts self‐disclosure of HIV status. Highlights specific threats like intimate partner violence and job dismissal as barriers to disclosure
Peltzer and Pengpid (2016)	Public health/behavioural medicine	Adult chronic disease patients, health facilities, Cambodia, Myanmar and Vietnam	Chronic illness (mixed)	Quantitative empirical (cross‐sectional survey)	Anticipated stigma (Chronic Illness Anticipated Stigma Scale [CIASS]; 12‐item multi‐item scale)	Reports prevalence (20.7%) of anticipated stigma in Southeast Asia. Links anticipated stigma to poorer health status (more chronic conditions, lower quality of life) and risky behaviour (smoking, poor diet). Finds healthcare workers are the largest source of anticipated illness stigma
Quinn and Chaudoir (2009)	Psychology/social psychology	College students living with concealable stigmatised identities (mixed), USA	Concealable stigmatised identities (CSI) (mixed)	Quantitative empirical (survey/structural equation modelling)	Anticipated stigma (anticipated stigma; 15‐item multi‐item scale explicitly asking likelihood of negative outcomes upon disclosure)	Proposes a framework detailing how anticipated stigma, identity centrality, and salience (intraindividual factors), plus cultural stigma (external factor), predict psychological distress. Anticipated stigma affects distress both directly and indirectly (via centrality and salience)
Quinn et al. (2014)	Psychology	Diverse, urban, adult community sample with CSI (mental illness, substance abuse, abuse/violence experiences), USA	Concealable stigmatised identities (CSI) (mixed)	Quantitative empirical (survey/regression analysis)	Anticipated stigma (anticipated stigma; 15‐item scale measuring concern about devaluation/mistreatment if identity becomes known)	Replicates finding that greater anticipated stigma predicts increased psychological distress in a diverse adult sample. Finds interaction: greater internalisation predicts distress primarily when centrality is high. Lower outness (concealment) also uniquely predicts increased distress
Quinn et al. (2015)	Psychiatric rehabilitation/psychology	Adults with mental illness (*N* = 105), urban community sample, USA	Mental illness stigma/internalised stigma	Quantitative empirical (survey/multiple serial mediation)	Anticipated stigma (anticipated stigma; 15‐item scale focusing on negative interpersonal reactions/devaluation, distinct from anticipated *discrimination*)	Experiences of discrimination lead to internalised stigma, fully mediated sequentially by increased anticipated discrimination and anticipated social stigma. Differentiates anticipated discrimination (acute acts) from anticipated stigma (day‐to‐day devaluation)
Quinn et al. (2020) (Visible & Concealable)	Psychology/social psychology	African American and Latinx adults reporting concealing a stigmatised identity (dual stigma/intersectionality focus), USA	Visible stigma (race/ethnicity) and concealable stigma (mixed)	Quantitative empirical (cross‐sectional regression/mediation)	Anticipated stigma (anticipated stigma of the CSI; 15‐item scale assessing perceived likelihood of mistreatment if CSI revealed)	Finds experienced racial discrimination predicts greater anticipated stigma of a CSI. The relationship between racial discrimination and depression is partially mediated by anticipated CSI stigma. Supports an additive model where both stigmas independently predict depression
Radusky et al. (2017)	Psychology/psychometrics	Adults living with HIV, Buenos Aires Metropolitan Area (AMBA), Argentina	HIV	Quantitative empirical (scale construction/pilot study)	Estigma Anticipado (anticipated stigma; part of a new HIV Stigma Inventory—IE‐VIH, 18 items)	Develops a new localised instrument (IE‐VIH). Finds higher levels of anticipated stigma than internalised or experienced stigma in the Argentine context
Reid (2020)	Sociology/cannabis studies	Medical and nonmedical cannabis users in the USA (with additional evidence from Canada and the UK) synthesised from qualitative social science studies	Cannabis‐related stigma and normalisation	Qualitative narrative review	Cannabis stigma, including structural, social and micro (felt/anticipated) stigma across multiple analytical levels	Synthesises qualitative literature to show cannabis stigmas persist despite legalisation, mapping structural, social, and individual forms of stigma and highlighting the concealable and intersectional nature of cannabis use, as well as anticipation‐driven concealment and normification strategies that complication normalisation narratives
Rice et al. (2019)	Public health/medicine/nursing	Older adult women living with HIV (WLWH), clinics in four US cities	HIV/ART adherence	Mixed methods (qualitative interviews + quantitative questionnaires)	Anticipated stigma in health care settings (12‐item scale/3 items specific to healthcare workers, adapted)	Anticipated stigma in healthcare settings is the only stigma measure significantly associated with suboptimal ART adherence. Identifies pathways: Anticipated stigma negatively impacts adherence via lower adherence self‐efficacy, higher depressive symptoms and coping by substance use
Tsai et al. (2021)	Global health/public health	Whole‐population survey (PLWH and HIV‐negative/unknown status), rural villages in southwestern Uganda	HIV/HIV stigma norms	Quantitative empirical (whole‐population survey/mediation analysis)	Perceptions of normative attitudes/anticipated stigma (omnibus score from 15‐item scale; wording adapted to perceive others' beliefs)	Finds participants commonly misperceive (overestimate) the pervasiveness of negative attitudes towards PLWH (pluralistic ignorance). Perceptions of norms (anticipated stigma) partially mediate the association between normative attitudes and personal attitudes
Turan et al. (2017)	Psychology/public health/behavioural health	People living with HIV (PLWH), HIV clinic (UAB, USA)	HIV/medication adherence/psychosocial outcomes	Quantitative empirical (survey/serial mediation/test of a framework)	Anticipated stigma (anticipated stigma by source: friends/family, community, healthcare workers; multi‐item scale adapted)	Tests a modified HSF, integrating perceived community stigma. Finds a serial mediation model where perceived community stigma—> internalised stigma—> anticipated community stigma—> lower medication adherence

*Note:* Table summarises the 31 empirical and conceptual sources that met inclusion criteria and directly informed the identification of attributes, antecedents, consequences and related constructs of anticipated stigma. An additional 34 foundational and contextual sources identified through citation chaining and prior integrative reviews contributed to the narrative synthesis but are not tabulated.

Abbreviations: ART, antiretroviral therapy; CIASS, Chronic Illness Anticipated Stigma Scale; COR, Conservation of resources; CSI, concealable stigmatised identity; HIVST, HIV self‐testing; IE‐VIH, Inventario de Estigma hacia personas con VIH (HIV Stigma Inventory); MSM, men who have sex with men; PLWH, people living with HIV; PrEP, pre‐exposure prophylaxis; SASS, Stigma and Self‐Stigma scale; TGW, transgender women; YLHIV, young people living with HIV.

### 3.4 Data Extraction and Analysis

For each included source, data were extracted into a structured matrix capturing: (1) disciplinary field and study design; (2) population and setting; (3) terminology used for anticipated stigma and related constructs; (4) explicit definitions or descriptions of anticipated stigma; (5) measurement approaches (where applicable); and (6) key findings related to attributes, antecedents, consequences and contextual factors (Table 1).

Consistent with Rodgers and Knafl's evolutionary method, analysis proceeded inductively and comparatively. Extracted data were repeatedly reviewed to identify recurring language and patterns that reflected potential defining attributes (e.g., future orientation, expectation of devaluation, disclosure‐linked nature), antecedents (e.g., intersectional identities, prior discrimination, legal and sociocultural context), and consequences (e.g., psychological distress, nondisclosure, altered healthcare engagement). Attention was also given to how anticipated stigma varies over time and across contexts within different conditions and disciplines, as well as how it overlaps with or differs from perceived, enacted and internalised stigma. These patterns were combined into a set of defining traits, causes and effects, which helped develop a conceptual model and a case example showing anticipated stigma related to disclosing cannabis use in nursing practice.

## 4 Overview of the Concept

### 4.1 Defining Attributes

Anticipated stigma is a future‐oriented expectation of negative treatment tied to concealable stigmatised identities, found across various disciplines with validated measurement tools. This type of stigma is associated with negative psychological, behavioural and health outcomes. It is studied from psychological, nursing, public health, sociological and organisational perspectives, focusing on social interactions, healthcare engagement, social norms, culture and workplace stigma. The defining attributes are listed in Table 2. Of note, while concealment is not listed as a defining attribute, anticipated stigma has been extensively studied in populations with concealable stigmatised identities, such as mental or chronic illness or substance use (Quinn and Chaudoir 2009; Quinn et al. 2014; King et al. 2024). Concealment is an important contextual factor or potential outcome of anticipated stigma.

**TABLE 2:** Defining attributes of anticipated stigma.

Attribute	Description	Cross‐disciplinary presence
Future‐oriented expectation	Anticipated stigma is defined by the expectation or fear of future negative treatment if a stigmatised identity or condition is revealed	Psychology, public health, nursing, social sciences
Social/interpersonal focus	Anticipated stigma centres on concerns about how others (family, friends, colleagues, healthcare workers, public) will react	All fields, with discipline‐specific emphasis (e.g., help‐seeking in nursing, symbolic interaction in sociology)
Emotional/cognitive anticipation	Involves worry, fear, or concern about being devalued, rejected, or discriminated against	Universal
Multidimensionality	Anticipated stigma is not monolithic; it varies by source, context and identity	All fields, with varying granularity
Link to social norms	Anticipated stigma is shaped by perceptions of prevailing social norms and cultural context	Public health, cross‐cultural psychology

*Note:* Fields include psychology, public health, nursing, social science, sociology, criminology, organisational studies and cross‐cultural studies.

The defining attributes of anticipated stigma are consistently supported across the literature and disciplines. It is inherently future‐oriented, involving the expectation of devaluation, discrimination, or rejection based on a stigmatised identity (Earnshaw et al. 2012; Quinn et al. 2014; Quinn and Chaudoir 2009; Tsai et al. 2021). Several studies also highlight its chronic nature and significance, conceptualising it as either a stable, trait‐like vulnerability or a dynamic, context‐sensitive process (Aslam and Sarwat 2021; Chaudoir and Quinn 2016; Moore and Tangney 2017). Psychologically, anticipated stigma acts as a stressor that can lead to negative outcomes (Chaudoir and Quinn 2016). Further, it frequently serves as a mediator or moderator between experienced discrimination and psychological distress, underscoring its theoretical and clinical significance (Quinn et al. 2015). Interpersonally, it is characterised by worries about negative reactions from others, especially in healthcare, work and family settings (Amarante et al. 2024; Earnshaw, Quinn, et al. 2013; Peltzer and Pengpid 2016; Rice et al. 2019). The construct is multi‐layered, changing based on social identity, source and setting (Earnshaw, Quinn, et al. 2013; Oga et al. 2022), and is influenced by wider cultural norms and societal expectations (Fujii and Hashimoto 2023; Ikizer et al. 2018; Tsai et al. 2021). Together, these insights derive the importance of context‐sensitive measurement and theory‐based intervention strategies.

### 4.2 Antecedents

Antecedents of anticipated stigma can be grouped into three main categories: individual factors, sociocultural influences and structural determinants. Among individual factors, sociodemographic variables such as age, gender, education level and minority status are consistently associated with greater anticipated stigma (Peltzer and Pengpid 2016; Quinn et al. 2020). Intersectional identities, such as being a transgender woman of colour living with HIV, may further heighten vulnerability to anticipated stigma through the compounding effects of multiple marginalised statuses (Amarante et al. 2024; Rice et al. 2019). Past experiences of discrimination, including enacted stigma, are also strongly associated with elevated anticipated stigma, reinforcing expectations of future mistreatment (Earnshaw, Quinn, et al. 2013; Quinn et al. 2015). Health‐related factors, including mental health symptom severity, comorbid conditions and the visibility of illness—particularly in chronic disease contexts—are similarly linked to higher levels of anticipated stigma (Earnshaw et al. 2012; Quinn et al. 2015). Identity‐related factors, especially the centrality and prominence of the stigmatised attribute with an individual's self‐concept, further shape anticipated stigma and influence its downstream effects on psychological distress and health outcomes (Quinn and Chaudoir 2009; Earnshaw, Quinn, et al. 2013). Finally, individual psychological traits, including coping strategies and resilience, moderate how anticipated stigma is experienced and managed (Quinn and Chaudoir 2009).

**FIGURE 1:**
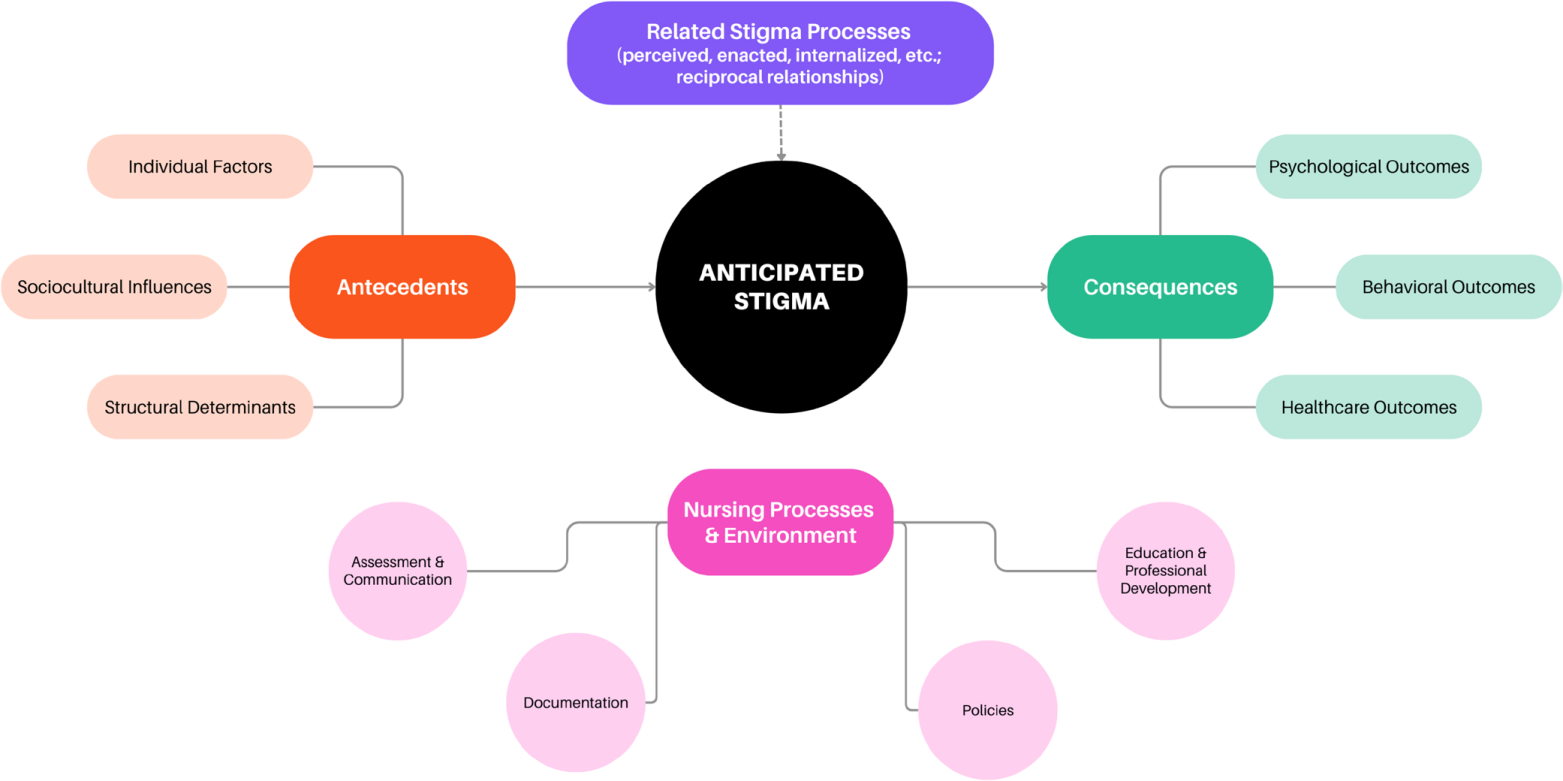
Conceptual model of anticipated stigma in nursing and cannabis use disclosure. Individual, sociocultural and structural antecedents influence anticipated stigma, which in turn shapes psychological, behavioural and healthcare outcomes. Related stigma processes (perceived, enacted and internalised stigma) and nursing processes and environments interact with anticipated stigma, mitigating or exacerbating its consequences over time.

Sociocultural influences shape anticipated stigma by establishing norms, expectations, and perceived social risks associated with identity and disclosure (Amarante et al. 2024). Cultural values, intersectional identities and close interpersonal relationships, especially with families and communities, can intensify fears of rejection or judgement (Quinn and Chaudoir 2009; Rice et al. 2019). Structural factors, including healthcare policies, legislation, media representations and institutional policies, further amplify these fears and vary substantially across sociopolitical contexts (Turan et al. 2017; Liu et al. 2020). For example, King et al. (2024) expressed that legal contradictions surrounding cannabis use, such as federal illegality despite state‐level legalisation, contribute to heightened fear of judgement and nondisclosure in healthcare settings. Institutional environments also play a reinforcing role: in the same study, only 15.1% initiated discussions about cannabis use, signalling structural silence that may perpetuate anticipated stigma. Social media exposure may further amplify anticipated stigma, whereas purposeful self‐disclosure and advocacy efforts can reduce it (Liu et al. 2020). Collectively, these sociocultural and structural forces function as key precursors that shape whether individuals expect stigmatising reactions during healthcare encounters. Figure 1 illustrates how individual, sociocultural and structural antecedents converge to shape anticipated stigma and its psychological, behavioural and healthcare‐related consequences in nursing contexts.

### 4.3 Consequences

The centrality and salience of the stigmatised identity influence how anticipated stigma affects psychological distress and health, with more central or salient identities amplifying its impact (Quinn and Chaudoir 2009; Quinn et al. 2015, 2020). Across chronic illness, HIV and mental health contexts, higher anticipated stigma is consistently associated with greater symptoms of depression, anxiety and stress, lower psychological well‐being and quality of life, and greater reliance on maladaptive coping strategies (Aslam and Sarwat 2021; Earnshaw et al. 2012; Earnshaw, Quinn, et al. 2013; Earnshaw, Smith, et al. 2013; Fujii and Hashimoto 2023; Peltzer and Pengpid 2016; Rice et al. 2019). Behaviorally, anticipated stigma has been linked to social withdrawal, disclosure avoidance, delays in testing and care, reduced adherence and counterproductive work behaviours (Aslam and Sarwat 2021; Follmer et al. 2022; Liu et al. 2020; Moore and Tangney 2017; Ngige and Ndayala 2020).

Cannabis‐related stigma provides a clear example of how legal and social context shape anticipated stigma. Legislative and shifting societal norms create regional differences in stigma, with stricter policies associated with greater perceived devaluation (King et al. 2024; Skliamis et al. 2022). Cannabis stigma has been linked to adverse psychological and physical outcomes (Ahern et al. 2007), and users often fear disbelief from providers regarding medical use, which undermines emotional well‐being and therapeutic engagement. Despite growing public acceptance, fear of judgement remains a significant barrier to disclosure in healthcare settings (Kerridge et al. 2017). Legal status contributes to perceived stigma, which influences internalised feelings and concealment behaviours, such as private use and odour masking (Hathaway et al. 2011; Lau et al. 2015; Skliamis et al. 2022). Even in regions where cannabis is more normalised, anticipated stigma can prompt nondisclosure, distancing and identity management strategies. For example, Bottorff et al. (2013) describe how cannabis remains a stigmatised treatment, particularly for patients with HIV/AIDS, chronic pain or mental health disorders. It is also important to distinguish stigma from legal concerns, as perceived legal risks can independently impede patient‐provider transparency (King et al. 2024; Lapham et al. 2022; Lau et al. 2015; Skliamis et al. 2022).

### 4.4 Related Concepts

To clarify the unique role of anticipated stigma as a concept, its importance lies in understanding what it is and what it is not (e.g., perceived, internalised, enacted stigma). Accordingly, this section identifies and distinguishes related constructs that are often conflated with or mistakenly used interchangeably with anticipated stigma. By mapping these boundaries, a more precise definition of the core attributes and role of anticipated stigma within broader stigma processes can be achieved.

#### 4.4.1 Perceived Stigma

Perceived stigma refers to an individual's current awareness or belief that others hold negative attitudes, labels or societal beliefs about a specific identity or behaviour, such as cannabis use (Kane et al. 2019; King et al. 2024; Skliamis et al. 2022; Zelaya et al. 2012). It is primarily a cognitive construct involving perceived devaluation and discrimination, which are often shaped by cultural narratives and healthcare dynamics, including labelling and stereotyping (King et al. 2024; Link 1987). In the context of cannabis use, individuals may expect judgement or misunderstanding from healthcare providers, leading to lower self‐esteem, avoidance of disclosure and delayed pursuit of care (Kerridge et al. 2017; King et al. 2024; Lau et al. 2015; Bottorff et al. 2013; LeBel 2008; Tesfaw et al. 2020). Importantly, perceived stigma often acts as a precursor to anticipated stigma, laying the groundwork for future beliefs about possible mistreatment (Stangl et al. 2019; Turan et al. 2017). While perceived stigma reflects current social judgements, it can also trigger future fears that influence behaviour and decision‐making, especially in stigmatised groups. As a variable factor, perceived stigma offers significant opportunities for intervention within provider–patient relationships and throughout the broader healthcare system (King et al. 2024; Troup et al. 2022).

#### 4.4.2 Internalised Stigma

Internalised stigma, also known as self‐ or felt stigma, is the process by which individuals absorb and accept negative societal beliefs about their stigmatised identity, integrating them into their self‐concept (Fernández et al. 2023; Kane et al. 2019; King et al. 2024; Livingston and Boyd 2010; Stangl et al. 2019; Turan et al. 2017). Unlike anticipated stigma, which is future‐oriented and involves expectations of upcoming judgement, internalised stigma is rooted in past and present experiences, manifesting as decreased self‐worth and changes in self‐identity (King et al. 2024; Livingston and Boyd 2010). In the context of cannabis use, this process reflects how individuals come to view themselves through the lens of societal stereotypes, especially regarding their perceived ability to fulfil regular roles at home or work (Hathaway et al. 2011). The cultural and legal status of cannabis, along with conflicting narratives about its benefits and harms, contribute to the development of internalised stigma. Social media analysis has shown that internalised stigma is one of the most common forms of cannabis‐related stigma, appearing in 38% of cannabis‐related posts (Chen et al. 2022). It influences attitudes, behaviours and disclosure practices among cannabis users, who may adjust their behaviour—such as hiding use or avoiding discussion—to conform to perceived social expectations (Hathaway et al. 2011).

#### 4.4.3 Enacted Stigma

Enacted stigma involves explicit experiences of discrimination or devaluation carried out by others, such as healthcare providers, peers, family members or broader social systems (Catona et al. 2016; Kane et al. 2019; King et al. 2024; Smith et al. 2016). This type of stigma is based on actual experiences and rooted in past or ongoing events, making it different from the future‐oriented nature of anticipated stigma. When it comes to cannabis use, enacted stigma might show up through being dismissed by healthcare providers, denied services, having appointments shortened or receiving lower‐quality care compared to non‐users (Ahern et al. 2007; King et al. 2024). These experiences are not only harmful in the moment but can also influence future decisions, contributing to internalised stigma and self‐criticism. Importantly, enacted stigma can directly lead to the development of anticipated stigma by reinforcing beliefs about being mistreated in the future.

#### 4.4.4 Additional Constructs

Several additional constructs help clarify the conceptual boundaries of anticipated stigma. *Symbolic interaction stigma* involves imagined social encounters and expectations of others' reactions, overlapping with but broader than anticipated stigma (Link et al. 2015). *Anticipated discrimination* focuses more narrowly on expected acts of exclusion, such as being denied care (Butt 2008; Khatooni 2023; Leurent and Ducasse 2023). *Stereotype threat* is a context‐specific fear of confirming negative stereotypes, often considered a subtype of anticipated stigma in healthcare and education settings (Thille et al. 2023). *Shame* reflects an emotional consequence of stigma, distinct from the anticipatory cognitive processes at the core of anticipated stigma (Leurent and Ducasse 2023). *Concealment* is a behavioural response to anticipated stigma, involving deliberate nondisclosure to avoid judgement, often accompanied by psychological costs (Cortopassi et al. 2024; King et al. 2024; Quinn and Chaudoir 2009). Lastly, *rejection sensitivity* increases vigilance towards exclusion and can influence the mental health effects of anticipated stigma (Cortopassi et al. 2024). A concise summary of the defining attributes, antecedents, outcomes and related concepts, along with illustrative examples and representative sources, is provided in Table 3.

**TABLE 3:** Summary of attributes, antecedents, consequences and related concepts of anticipated stigma.

Component	Brief definition/description	Key examples	Representative sources
Attributes	Future‐oriented expectation of devaluation; social/interpersonal focus, etc.	Fear of judgement by providers; concern about rejection	Earnshaw et al. (2012), Quinn and Chaudoir (2009)
Antecedents	Individual, sociocultural and structural precursors	Intersectional identities; prior discrimination; legal context of cannabis	Amarante et al. (2024), King et al. (2024), Turan et al. (2017)
Consequences	Psychological and behavioural outcomes	Depressive symptoms; nondisclosure; reduced utilisation	Aslam and Sarwat (2021), Rice et al. (2019)
Related Concepts	Constructs often conflated with anticipated stigma, clarified in this analysis	Perceived stigma; internalised stigma; enacted stigma; concealment; stereotype threat	Earnshaw and Chaudoir (2009), Kane et al. (2019), Livingston and Boyd (2010), Smith et al. (2016)

### 4.5 Model Case

Jasmine, a 46‐year‐old woman scheduled for elective surgery, visits the preoperative clinic for assessment. She regularly uses cannabis to manage anxiety and chronic pain, having researched its benefits online and through dispensary staff due to difficulty discussing it with healthcare providers. Living in a state where cannabis remains controversial despite legal medical use, she is aware of societal stigma towards cannabis users in healthcare, especially as a middle‐aged woman with chronic conditions. During medication reconciliation, Jasmine employs a selective disclosure strategy she has developed over time. She discusses her prescription medications but provides only partial information about her cannabis use, carefully watching the nurse's reactions before revealing more. She fears being labelled ‘drug‐seeking’, judged, or having her pain dismissed. She also fears future impacts on her record and insurance. When the nurse seems rushed and uncomfortable discussing cannabis, Jasmine adopts defensive tactics, emphasising her responsible use, medical necessity and compliance with laws, while internally questioning full disclosure. Though she has not faced negativity in this encounter, her past experiences and societal attitudes have made her cautious. Ultimately, she discloses minimal information, acknowledging that limited provider knowledge and discomfort hinder meaningful discussions. This partial disclosure risks damaging the therapeutic relationship and could lead to drug interactions or inadequate pain management. This case illustrates how anticipated stigma, provider gaps and documentation concerns impede optimal care and patient safety.

## 5 Discussion

Anticipated stigma is an evolving concept with increasing importance across healthcare fields, yet it remains underrepresented within nursing science. Based on the expectation of future devaluation, judgement, or discrimination, it is especially relevant for individuals with concealable stigmatised identities, such as cannabis users. Although interdisciplinary research—particularly in HIV, mental health and chronic illness—has recognised anticipated stigma as a significant barrier to disclosure and care engagement, its relevance to cannabis use in nursing settings is just beginning to be explored. Current research, including studies such as King et al. (2024), suggests that anticipated stigma is a strong predictor of non‐disclosure and contributes to poorer clinical outcomes. Nonetheless, the concept is often mixed with related forms of stigma (e.g., perceived, internalised and enacted), and measurement tools lack clear conceptual foundations, which limits their applicability across different conditions and environments.

The findings refine existing stigma frameworks by clarifying the temporal and anticipatory dimensions of stigma in nursing contexts. Whereas Link and Phelan's (2001) framework emphasises labelling, stereotyping, and status loss rooted in power, minority stress models highlight cumulative exposure to prejudice, anticipated stigma captures the prospective, scenario‐based calculations that individuals make when deciding whether to disclose stigmatised identities or behaviours such as those associated with cannabis use. By synthesising evidence from psychology, public health, sociology and organisational studies, this analysis reveals that anticipated stigma functions as a key mechanism linking structural and interpersonal stigma to downstream psychological and behavioural outcomes. For nursing, this highlights the importance of not only addressing past experiences of discrimination but also the expectations that patients bring into clinical encounters about how nurses and other providers will respond.

### 5.1 Implications for Nursing Practice and Institutional Policy

Anticipated stigma has direct implications for everyday nursing practice and institutional policies. At the point of care, nurses can either reinforce or mitigate anticipated stigma through their approach, communication style, non‐verbal cues and documentation practices. For example, integrating nonjudgmental, behaviour‐specific prompts (e.g., ‘Aside from prescription medications, many patients use cannabis, herbs, or other supplements to manage symptoms. Do you use any of these that we should know about to help keep you safe?’) into nursing assessments may normalise disclosure and signal psychological safety. Institutionally, nursing leaders can advocate for stigma‐informed documentation policies that avoid pejorative labels, clarify the clinical rationale for recording cannabis use or other stigmatised behaviours, and limit unnecessary dissemination of sensitive information across the record (Akatukwasa et al. 2021; King et al. 2024). Policies that promote patient‐centred language, accuracy, confidentiality and shared decision‐making may reduce anticipated stigma and facilitate transparent discussions about risks and benefits (Amarante et al. 2024; Bottorff et al. 2013). The perioperative environment is one example of a specialised setting where these approaches are particularly important for safe medication reconciliation, anaesthesia planning, harm reduction and postoperative pain management for patients who use cannabis (King et al. 2024).

### 5.2 Implications for Nursing Education and Professional Development

This concept analysis also emphasises the importance of including anticipated stigma in nursing curricula and ongoing professional education. Educational programs can seek to explain how anticipated stigma functions, including its roots in systemic inequities and its effects on disclosure, trust and health outcomes (Amarante et al. 2024; King et al. 2024; Quinn et al. 2020). Simulation and case‐based learning, using scenarios such as the model case presented here, can help students and clinicians practice responding to stigmatised disclosures with empathy, curiosity, ethics and clinical precision. Additionally, incorporating stigma‐informed communication skills like reflective listening, validation of patient concerns and collaborative risk–benefit discussions into competency frameworks can help develop nurses into stigma‐sensitive clinicians. Faculty and preceptors may also benefit from training that encourages reflection on their own assumptions about substance use, chronic illness and other stigmatised conditions to reduce unintended contributions to anticipated stigma in clinical learning environments (Bottorff et al. 2013; Hulaihel et al. 2023; Rice et al. 2019).

### 5.3 Implications for Research

The attributes, antecedents and consequences identified in this analysis point to several priorities for future nursing research. First, there is a need for condition‐specific, psychometrically robust, and theoretically grounded measures of anticipated stigma that are anchored in clear definitions and sensitive to diverse sociocultural and legal contexts, including substance use and cannabis. Although King et al. (2024) indicate that anticipated stigma is strongly linked to cannabis nondisclosure, this represents an emerging area rather than a mature body of work. Qualitative and mixed‐methods studies should explore how patients and nurses jointly construct and negotiate anticipated stigma in real‐time encounters, and how provider communication, institutional policies, and relational care dynamics shape the anticipation of stigma in critical interactions (e.g., nursing intake and assessment encounters). Longitudinal research is needed to examine how anticipated stigma evolves across the care trajectory and to clarify its temporal relationships with perceived, enacted and internalised stigma. Finally, intervention studies should test stigma‐informed nursing approaches, such as cannabis‐specific disclosure prompts, tailored assessment questions, communication training and documentation reforms, to evaluate their effects on anticipated stigma, disclosure, psychological safety and downstream clinical outcomes, thereby informing stigma‐responsive models of nursing care and health equity. In particular, cannabis use serves as a key example for studying anticipated stigma in nursing: it is becoming more common and often legal, yet it remains morally debated, poorly documented, and frequently undisclosed, making it an ideal context for developing and testing stigma‐aware nursing interventions (King et al. 2024).

### 5.4 Limitations

This concept analysis has several limitations. First, despite conducting an interdisciplinary search, the sources included were mainly identified using an AI‐assisted semantic tool (Elicit), along with traditional database searches and citation chaining. This method is suitable for the iterative, exploratory nature of evolutionary concept analysis but is limited by the coverage and ranking algorithms of the underlying databases and may be difficult to reproduce precisely. Second, although a small number of non‐English sources were translated, most of the sample still came from peer‐reviewed literature. It might not fully represent work published in other languages or in grey literature. Third, most empirical research on anticipated stigma focuses on HIV, mental illness, or chronic illness, with only early evidence related to cannabis use, which could restrict applicability to this emerging area and other contexts. Fourth, the reliance on cross‐sectional, self‐report studies limits the ability to draw conclusions about causal relationships and changes over time. Finally, while the model case and implications are grounded in nursing, they may need adaptation to fit local legal and organisational environments.

## 6 Conclusion

This concept analysis clarifies anticipated stigma as a future‐oriented expectation of devaluation or discrimination, distinct from perceived, internalised and enacted stigma, yet closely connected with them across time and context. By synthesising interdisciplinary evidence and applying it to cannabis use disclosure in nursing, the analysis provides a theoretically grounded, nursing‐specific understanding of how anticipated stigma undermines therapeutic communication, psychological safety and equitable care. The attributes, antecedents and consequences identified here, along with the proposed conceptual model, offer a foundation for developing stigma‐informed assessment tools, educational interventions and organisational policies. This work contributes to ongoing efforts in nursing science to recognise stigma as a structural determinant of health and to create stigma‐informed, person‐centred models of care.

## Funding

The author has nothing to report.

## Ethics Statement

The author has nothing to report.

## Conflicts of Interest

The author declares no conflicts of interest.

## Supporting information


**Data S1:** jan70499‐sup‐0001‐DataS1.docx.

## Data Availability

The author has nothing to report.
