# Supplementary material for: Anticipated Stigma in Nursing: A Concept Analysis Informed by Cannabis Use Disclosure
Source: J Adv Nurs. 2026 Jan 26;82(9):8510–30. doi: 10.1111/jan.70499 (PMC13460993; doi:10.1111/jan.70499)
Supplement: Supplementary file 1 [file JAN-82-8510-s001.docx]

|  | **Standards for Reporting Qualitative Research (SRQR)*** |  |
| --- | --- | --- |
|  | <http://www.equator-network.org/reporting-guidelines/srqr/> |  |
|  |  | **Page/line no(s).** |
| **Title and abstract** | |  |
|  | **Title** - Concise description of the nature and topic of the study Identifying the study as qualitative or indicating the approach (e.g., ethnography, grounded theory) or data collection methods (e.g., interview, focus group) is recommended | **Yes –** Title identifies the study as a concept analysis and clearly states the phenomenon of interest (“Anticipated Stigma in Nursing: A Concept Analysis Informed by Cannabis Use Disclosure”). **Location:** Page 1, Line 1. |
|  | **Abstract** - Summary of key elements of the study using the abstract format of the intended publication; typically includes background, purpose, methods, results, and conclusions | **Yes –** Structured abstract includes aim, design, data sources, review methods, results, conclusion, and impact, followed by a note on patient/public contribution and key words. **Location:** Page 1, Lines 3–23; Page 2, Lines 24 & 26–27. |
| **Introduction** | |  |
|  | **Problem formulation** - Description and significance of the problem/phenomenon studied; review of relevant theory and empirical work; problem statement | **Yes –** Introduction and Background describe anticipated stigma as a future-oriented expectation of prejudice, devaluation, or discrimination; summarize interdisciplinary theory and frameworks (e.g., Goffman, Minority Stress, Conservation of Resources); and identify the gap in nursing scholarship and relevance to cannabis disclosure. **Location:** Page 3, Lines 49–67; Page 4, Lines 70–76; Page 5, Lines 100–114; Page 6, Lines 115–124, 126–137. |
|  | **Purpose or research questio**n - Purpose of the study and specific objectives or questions | **Yes –** Purpose is clearly stated in the abstract and reiterated at the end of the introduction: to clarify the concept of anticipated stigma using Rodgers and Knafl’s evolutionary concept analysis and to examine its relevance to cannabis use disclosure and nursing practice. **Location:** Page 1, Lines 3–6; Page 4, Lines 73–76. |
| **Methods** | |  |
|  | **Qualitative approach and research paradigm** - Qualitative approach (e.g., ethnography, grounded theory, case study, phenomenology, narrative research) and guiding theory if appropriate; identifying the research paradigm (e.g., postpositivist, constructivist/ interpretivist) is also recommended; rationale** | **Yes –** A concept analysis of anticipated stigma is conducted using Rodgers and Knafl’s (2000) evolutionary method, emphasizing concepts as dynamic and context-dependent and detailing six iterative strategies (identifying the concept, sampling literature, extracting data, analyzing and summarizing, identifying a model case, and proposing implications). This interpretive, context-sensitive approach aligns with an interpretivist/constructivist paradigm and is explicitly linked to SRQR/EQUATOR guidance. **Location:** Page 9, Lines 189–196; Page 9–10, Lines 197–206; Page 10, Lines 207–213. |
|  | **Researcher characteristics and reflexivity** - Researchers’ characteristics that may influence the research, including personal attributes, qualifications/experience, relationship with participants, assumptions, and/or presuppositions; potential or actual interaction between researchers’ characteristics and the research questions, approach, methods, results, and/or transferability | **N/A** |
|  | **Context** - Setting/site and salient contextual factors; rationale** | **N/A** |
|  | **Sampling strategy** - How and why research participants, documents, or events were selected; criteria for deciding when no further sampling was necessary (e.g., sampling saturation); rationale** | **Yes –** A purposive, interdisciplinary sampling strategy is described: iterative semantic searches using Elicit; supplementary keyword searches in PsycINFO and CINAHL; citation chaining; and integration of seminal sources from prior integrative reviews. Inclusion and exclusion criteria for empirical, theoretical, and concept-analytic sources are detailed, and the final sample is defined as 31 core sources plus 34 additional conceptual/theoretical sources spanning 1963–2024. **Location:** Page 10–11, Lines 215–223 and 230–250; “Eligibility Criteria and Study Selection,” Lines 236–247. |
|  | **Ethical issues pertaining to human subjects** - Documentation of approval by an appropriate ethics review board and participant consent, or explanation for lack thereof; other confidentiality and data security issues | **N/A** |
|  | **Data collection methods** - Types of data collected; details of data collection procedures including (as appropriate) start and stop dates of data collection and analysis, iterative process, triangulation of sources/methods, and modification of procedures in response to evolving study findings; rationale** | **Yes –** Data collection consisted of iterative literature searching and selection procedures: AI-assisted semantic searches with Elicit across a large corpus; supplementary database searches; citation chaining; and integration of sources from prior integrative reviews. The process reflects multiple methods (AI-assisted search, traditional databases, citation chaining) and disciplines and is explicitly tied to the iterative nature of Rodgers and Knafl’s evolutionary method. **Location:** “Search Strategy and Data Sources,” Page 10–11, Lines 215–223 and 230–234; “Eligibility Criteria and Study Selection,” Lines 236–247. |
|  | **Data collection instruments and technologies** - Description of instruments (e.g., interview guides, questionnaires) and devices (e.g., audio recorders) used for data collection; if/how the instrument(s) changed over the course of the study | **Yes –** Data collection technologies are identified, including Elicit (an AI-supported research assistant performing semantic searches across large bibliographic corpora) and Zotero for reference management and screening. The manuscript also acknowledges the use of generative AI tools for organization and refinement of manuscript language and structure, with the author retaining final responsibility for content. **Location:** Page 10, Lines 215–223 (Elicit); Page 10–11, Lines 224–227 and 230–233 (Zotero and supplemental searching); Page 4, Lines 75–79 (generative AI support statement). |
|  | **Units of study** - Number and relevant characteristics of participants, documents, or events included in the study; level of participation (could be reported in results) | **Yes –** Units of study are the 31 included empirical and conceptual sources directly contributing to the concept analysis, plus 34 additional sources incorporated for conceptual and theoretical clarification. Characteristics (disciplinary field, population/setting, terminology, definitions, measurement, and key findings) are captured in a structured data extraction matrix and summarized in Table 1. **Location:** Page 11, Lines 243–247; Page 12, Lines 252–256; Table 1. |
|  | **Data processing** - Methods for processing data prior to and during analysis, including transcription, data entry, data management and security, verification of data integrity, data coding, and anonymization/de-identification of excerpts | **Yes –** For each included source, data were extracted into a structured matrix capturing disciplinary field, population and setting, terminology, explicit definitions, measurement approaches, and key findings related to attributes, antecedents, consequences, and contextual factors. This matrix served as the basis for subsequent inductive comparison and synthesis. **Location:** Page 12, Lines 252–256. |
|  | **Data analysis** - Process by which inferences, themes, etc., were identified and developed, including the researchers involved in data analysis; usually references a specific paradigm or approach; rationale** | **Yes –** Data analysis proceeded inductively and comparatively in line with Rodgers and Knafl’s evolutionary method, with repeated review of the extraction matrix to identify recurring language and patterns, develop defining attributes, antecedents, and consequences, and examine contextual and temporal variation and overlaps with perceived, enacted, and internalized stigma. These patterns informed construction of a conceptual model and a model case illustrating anticipated stigma in nursing practice. **Location:** Page 12, Lines 257–267; “Overview of the Concept” and “Defining Attributes,” Page 12–14, Lines 268–299. |
|  | **Techniques to enhance trustworthiness** - Techniques to enhance trustworthiness and credibility of data analysis (e.g., member checking, audit trail, triangulation); rationale** | **Yes –** Trustworthiness is supported by inclusion of interdisciplinary empirical and theoretical sources; transparent description of AI-assisted and traditional search strategies; use of a structured data extraction matrix; development of a conceptual model (Figure 1) and detailed model case; and explicit discussion of methodological limitations (e.g., dependence on Elicit’s coverage and ranking algorithms, language and publication-type constraints). These techniques enhance transparency, conceptual saturation, and transferability. **Location:** Page 9–10, Lines 189–206; Page 10–12, Lines 215–267; Figure 1 and accompanying note, Page 16, Lines 334–342; “Limitations,” Page 25, Lines 533–541. |
| **Results/findings** | |  |
|  | **Synthesis and interpretation** - Main findings (e.g., interpretations, inferences, and themes); might include development of a theory or model, or integration with prior research or theory | **Yes –** The analysis synthesizes anticipated stigma as a future-oriented expectation of negative treatment associated with concealable stigmatized identities (e.g., cannabis use), identifies defining attributes, antecedents, consequences, and distinguishing features relative to perceived, enacted, and internalized stigma, and develops a conceptual model and model case specific to nursing and cannabis disclosure. Findings are integrated with foundational stigma frameworks (e.g., Goffman, Link & Phelan, Earnshaw et al.) and contemporary minority stress and structural stigma literature. **Location:** “Overview of the Concept” and “Defining Attributes,” Page 12–14, Lines 268–299; “Antecedents” and “Consequences,” Page 14–17, Lines 300–349; “Related Concepts,” Page 18–20, Lines 372–434; conceptual model (Figure 1), Page 16, Lines 334–342; model case and lead-in to Discussion, Page 22, Lines 453–456. |
|  | **Links to empirical data** - Evidence (e.g., quotes, field notes, text excerpts, photographs) to substantiate analytic findings | **Yes –** The manuscript uses multiple forms of evidence to substantiate analytic findings: Table 1 summarizes characteristics of the 31 included sources; Table 2 lists defining attributes across disciplines; Table 3 summarizes attributes, antecedents, consequences, and related concepts with key examples and representative sources; the conceptual model (Figure 1) is grounded in cited literature; and the model case narrative is informed by empirical findings on cannabis disclosure in nursing. **Location:** Table 1; Table 2, Page 13, Lines 280–283; Table 3, Page 20–21, Lines 433–437, 49–56; Figure 1 and note, Page 16, Lines 334–342; model case, Page 22, Lines 453–456. |
|  |  |  |
| **Discussion** | |  |
|  | **Integration with prior work, implications, transferability, and contribution(s) to the field -** Short summary of main findings; explanation of how findings and conclusions connect to, support, elaborate on, or challenge conclusions of earlier scholarship; discussion of scope of application/generalizability; identification of unique contribution(s) to scholarship in a discipline or field | **Yes –** The Discussion clarifies how anticipated stigma refines and extends existing stigma frameworks by emphasizing temporal and anticipatory dimensions, links structural and interpersonal stigma to downstream psychological and behavioral outcomes, and situates findings within nursing contexts, especially around cannabis disclosure. Subsequent sections articulate implications for nursing practice and institutional policy, nursing education and professional development, and future research, highlighting the concept’s transferability to other stigmatized health behaviors and its unique contribution to nursing theory and stigma-informed care. **Location:** “Discussion,” Page 22–23, Lines 457–479; “Implications for Nursing Practice and Institutional Policy,” Page 23, Lines 481–497; “Implications for Nursing Education and Professional Development” and “Implications for Research,” Page 24–25, Lines 499–532. |
|  | **Limitations** - Trustworthiness and limitations of findings | **Yes –** The Limitations section acknowledges that the search relied heavily on an AI-assisted semantic tool (Elicit) and available databases, which may introduce coverage and ranking biases and limit reproducibility; that most sources were peer-reviewed and predominantly English-language, potentially underrepresenting non-English and grey literature; and that the conceptual, literature-based nature of the work constrains direct generalizability while still offering transferable insights. These constraints are framed as appropriate but important to consider for interpretation and future research. **Location:** “Limitations,” Page 25, Lines 533–541 (and following sentences as applicable). |
|  |  |  |
| **Other** | |  |
|  | **Conflicts of interest** - Potential sources of influence or perceived influence on study conduct and conclusions; how these were managed | **Yes –** A conflict of interest statement is provided with the manuscript materials; the author declares no conflicts of interest that could influence the conduct, interpretation, or reporting of the study. **Location:** Title page / author declarations (separate from the line-numbered main text).  Completed_SRQR_Checklist |
|  | **Funding** - Sources of funding and other support; role of funders in data collection, interpretation, and reporting | **Yes –** A funding statement accompanying the manuscript indicates that no external funding was received and that no funder influenced the study’s design, analysis, or reporting. **Location:** Title page / author declarations (separate from the line-numbered main text). |
|  |  |  |
|  | *The authors created the SRQR by searching the literature to identify guidelines, reporting standards, and critical appraisal criteria for qualitative research; reviewing the reference lists of retrieved sources; and contacting experts to gain feedback. The SRQR aims to improve the transparency of all aspects of qualitative research by providing clear standards for reporting qualitative research. |  |
|  |  |  |
|  | **The rationale should briefly discuss the justification for choosing that theory, approach, method, or technique rather than other options available, the assumptions and limitations implicit in those choices, and how those choices influence study conclusions and transferability. As appropriate, the rationale for several items might be discussed together. | **Justification:** Rodgers and Knafl’s evolutionary concept analysis method was selected because it emphasizes inductive, iterative analysis of how concepts evolve over time and across disciplines, supporting an interpretivist stance that recognizes contextual and temporal variation in how anticipated stigma is experienced and conceptualized. This rationale is discussed in the Methods section in connection with the multi-theoretical frameworks reviewed earlier. **Updated location:** Page 9–10, Lines 189–206. |
|  |  |  |
|  | **Reference:** |  |
|  | O'Brien BC, Harris IB, Beckman TJ, Reed DA, Cook DA. **Standards for reporting qualitative research: a synthesis of recommendations.** *Academic Medicine*, Vol. 89, No. 9 / Sept 2014  DOI: 10.1097/ACM.0000000000000388 |  |
|  |  |  |
|  |  |  |
